# Relevance of social contact definitions for use in infectious disease transmission modeling: a systematic review and recommendations

**DOI:** 10.1186/s12879-026-12938-y

**Published:** 2026-03-18

**Authors:** Charlotte Doran, Mahmud Sheku, Yukiko Nakada, Ben Lopman, Kristin Nelson

**Affiliations:** Emory University Rollins School of Public Health, 1518 Clifton Road, Atlanta, GA 30322 USA

**Keywords:** Mixing patterns, Social contacts, Infectious disease dynamics, Mathematical modeling, Pathogen transmission

## Abstract

**Background:**

Social mixing studies provide crucial evidence for parameterizing mathematical models of infectious disease transmission, and their validity for use in models is dependent on their study design and how well the contacts they capture map to the transmission routes of the pathogen of interest. This systematic review aims to catalogue contact definitions used in social mixing studies from 2005 to 2024 and evaluate their conceptual alignment to three archetypal pathogens.

**Methods:**

We searched Ovid Medline, Embase, Scopus, and Global Index Medicus for studies of human-to-human interactions that collected data via survey, diary, or interview published between January 2005 and August 2024. We excluded studies that focused on sexually-transmitted or food/water/vector-borne pathogens or used only GPS or sensor data. We excluded studies of contact tracing as a public health effort. Screening and data collection were conducted in Covidence. Contact definitions were presented verbatim and qualitatively coded to identify key elements. Results were stratified by prospective or retrospective design. We used the Mixed Methods Appraisal Tool to evaluate risk of bias.

**Results:**

We included 112 eligible studies, half (52.7%) of which began during pandemic periods (H1N1 [2009] or COVID-19 [2020]), commonly in the United States (18%) or United Kingdom (16%). Most (68%) had a retrospective design in which participants were asked to recall contacts that occurred prior to survey administration and 73% used a single-survey cross sectional design using random (16%) or stratified random (44%) sampling. Relevant contacts were often defined by an exchange of words (77%) or physical touch (59%). A minority of studies differentiated between contacts that occurred outdoors vs. indoors (28%) or allowed participants to report large group contacts separately from individually named contacts (28%). Contact definitions and attributes conceptually aligned with the transmission biology of influenza, tuberculosis, and norovirus in 77%, 6%, and 50% of studies respectively.

**Conclusions:**

Contact studies were limited in their global scope and non-pandemic representativeness. Critical modifiers of transmission risk such as location, household membership, and shared space with large numbers of people were under-measured. Future modeling and social mixing studies should align measured contact rates to the target transmission routes by incorporating these elements.

**Supplementary Information:**

The online version contains supplementary material available at 10.1186/s12879-026-12938-y.

## Introduction

Social mixing studies in the context of infectious disease are a unique set of study designs that ask participants to recall, over a specified period of time, the number and characteristics of individuals with whom they have person-to-person interactions. These data collection efforts are vital sources of empirical data on contact patterns that are used to parameterize mathematical models of infectious disease. They can provide information on contact frequency, where contacts occur, and who interacts with whom. In models, these data inform relevant clinical and demographic strata (e.g. age, sex, and comorbidities) and assortativity (tendency towards “like with like” contacts or interactions between people with shared characteristics) in mixing patterns, which determine how infections spread. For each contact, the probability of transmission given contact depends on a variety of biological and environmental factors, including the pathogen of interest and its minimum infectious dose, host infectiousness, contact susceptibility, and the length and location of exposure [1]. As a result, the conceptual alignment of study definitions of “contact” with known transmission biology can impact the validity of using these data to model transmission dynamics.

The POLYMOD study evaluated social mixing and contact patterns relevant to transmission of close-contact infections in eight countries in Europe between May 2005 and September 2006. POLYMOD defined a relevant contact as “either skin-to-skin contact such as a kiss or handshake (a physical contact), or a two-way conversation with three or more words in the physical presence of another person but no skin-to-skin contact (a nonphysical contact).” [2] Since the initial publication of its results in 2008, this study has been used to parameterize many models of infectious disease transmission [3–6], and its contact definition is commonly used or adapted for new social mixing studies in other populations [7–10]. While this contact definition was designed to capture the most epidemiologically relevant encounters for both respiratory droplet and close contact transmission, it is limited by its focus on conversational or physical contact, which may not be necessary for transmission of some types of pathogens. While analyses have shown POLYMOD data fits well to transmission of varicella and parvovirus B19 [11], transmission of other airborne pathogens like tuberculosis and influenza is not well characterized by touch or close-range contacts alone [12, 13]. While prior systematic reviews of contact studies have evaluated differences in observed contact patterns or definitions used [14–16], few reviews have explicitly evaluated the biological relevance of contact definitions to the actual transmission process of specific pathogens.

In addition to which data contacts surveys capture, the design of the study and survey itself can have meaningful impacts on data validity. In retrospective contact surveys where participants recall contacts that occurred in the past over a specified recall period, participants tend to report higher-intensity contacts or contacts with individuals that are more familiar to the participant, which can mischaracterize transmission pathways for some pathogens. For example, casual or community contacts that are not recalled may disproportionately bias contact estimates for highly infectious pathogens transmitted through the air, but less so for pathogens that require extended durations of shared space. Prospective designs, where participants record contacts as they happen over the course of a reporting period (e.g. through a ‘diary’, or record of contacts), can alleviate some of these limitations, but can also suffer from survey fatigue bias, especially for surveys with a long reporting period or longitudinal follow-up [17]. Systematic underestimates of contact rates at particular times of year can skew model outputs for seasonally transmitted pathogens such as flu and norovirus. Since POLYMOD was conducted, electronic surveys have become substantially more accessible, making reporting of contacts in real time faster and easier for participants. Electronic contact surveys were key during the COVID-19 pandemic when in-person interactions were substantially restricted, and quick deployment was critical to understand transmission patterns and impacts of social distancing restrictions, but these surveys, naturally, focused on contacts relevant to SARS-CoV-2 transmission, making transportability to other pathogens difficult [18]. 

In this review, we define three modes of transmission for respiratory pathogens, in accordance with recent World Health Organization (WHO) categorization [19]: airborne transmission, in which particles are suspended in the air and are inhaled; direct deposition, where particles travel short distances in a semi-ballistic trajectory before landing in the eyes, nose, or mouth; and direct contact, in which pathogens are transmitted through skin-to-skin contact. Because social mixing data are not routinely used to model pathogens transmitted primarily by indirect contact (transmitted via an intermediate surface) which does not require human-to-human interaction, we did not include it in our consideration. The objectives of this systematic review are to (1) catalogue how survey-based studies of social mixing have defined epidemiologically relevant human-to-human contacts since 2005, including key features of a contact definition such as duration, distance, and other criteria (i.e. touch, exchange of words) (2), understand the potential for bias stemming from survey and study design, and (3) evaluate how well these definitions structurally align with transmission biology for three commonly-modeled pathogens that will serve as a framework for the WHO-defined transmission routes against which to evaluate studies: influenza (primarily direct deposition, some airborne), tuberculosis (airborne), and norovirus (direct contact). Finally, this review provides recommendations for the use of existing contact data for model parameterization and for the design of future social mixing studies.

## Methods

### Protocol and reporting

This systematic review was developed using the Preferred Reporting Items for Systematic reviews and Meta-Analyses (PRISMA) guidelines for systematic review protocols (PRISMA-P) and reporting. Prior to data collection, we registered the protocol for this review on PROSPERO/OSF – ID: CRD42025642538. After registration of the protocol, technical difficulties arose that prevented the planned use of AI-assisted systematic review data collection.

### Search strategy

The search strategy was adapted from previous reviews of social mixing studies [15, 18]. We queried Ovid Medline, Embase, Scopus, and Global Index Medicus for studies published on or after January 1, 2005 up until August 30, 2024 (the date the search was conducted) in English using the following search string, adapted as needed for each database:

((contact adj2 (rate* OR study OR studies OR pattern* OR histor* OR network*)) OR (mixing adj2 (study OR studies OR pattern* OR social* OR behavio?r*))) AND (survey* OR questionnaire* OR diary OR diaries)

The full search strategy for each database can be found in the Supplemental material 2 (Appendix A). All results were imported into Covidence software for screening and data extraction.

### Eligibility criteria

Eligibility criteria for social mixing studies were adapted from Hoang et al. [15] and included only those with full text available in English that focused on social mixing patterns relevant to the spread of respiratory or enteric infections. Studies were considered eligible if they met the following inclusion criteria: (1) primary focus on human-to-human interactions (“contacts”) or shared physical space with infectious disease transmission potential, implying the physical presence of at least two people (2), data collection via survey, diary, or interview, either online/electronically, on paper, in person, or by phone (3), participants were asked to record all contacts that occurred over a specified reporting period and setting, and (4) collected data on or after 2005.

We excluded studies that: (1) focused solely on sexual contacts or human-animal/animal-animal contacts only (2), had a primary focus on infections transmitted by food/water reservoirs or vectors (3), relied solely on cell phone records, mobility, or sensor data to define relevant contacts, and (4) collected data only for the purpose of contact tracing of cases or collected solely the history of contact with an infectious case. We defined contact tracing studies as efforts involving identification of symptomatic individuals and their contacts, with the index case interview defining eligibility of others (contacts) for the study. Social mixing studies define a source population at baseline and enroll eligible individuals via a pre-defined sampling mechanism. Studies with overlapping datasets were excluded.

### Screening and data collection

We used Covidence for all screening and data collection. Duplicates were removed using a two-step workflow: automatic exact matching on DOI and titles by Covidence, followed by manual review using title matching plus author and year of publication. For preprint-publication pairs, we retained the peer-reviewed article. Title and abstract screening was conducted independently by co-authors CD, YN and MS, with each article screened by two reviewers and conflicts resolved by discussion and final vote by CD. Similarly, data extraction was done by consensus between at least two of CD, YN, and MS for all elements.

### Data items

The study setting, eligibility criteria, timeframe of data collection, study design, sampling strategy, survey format, sample size, and relation of survey respondent to participant were extracted onto custom Covidence templates. The data extraction template used for this review can be found in the supplementary material (Appendix B). We recorded the verbatim contact definition used in the instrument, as well as whether the survey (1) allowed large group contacts to be input separately from named individual contacts (2), differentiated between indoor vs. outdoor, household vs. non-household, and physical touch vs. non-touch contacts, or (3) captured distance and duration of the interaction. If the number of contacts allowed to be reported by a single participant was limited, either by the survey instrument or in the analysis, we recorded the maximum allowed number of contacts. The estimated contact rate and measures of variability were collected and presented in the supplementary material along with their corresponding contact definitions and pathogen alignment Supplementary file 4 (Appendix C). Combined measures of contact rates are not presented due to heterogeneity of contact results. As survey questions were often included in publications as appendices, supplementary material was also reviewed for data extraction if information was unavailable in the main text.

To inform the data collected for this review, we also conducted an unstructured literature review to characterize the transmission routes of influenza, tuberculosis, and norovirus and mapped the information to applicable elements of the contact definition and characteristics. These three pathogens were chosen based on their well-characterized transmission biology and their specificity to WHO-defined transmission routes. In addition, we aimed to include at least one viral and at least one bacterial pathogen. The three example pathogens are not intended to be representative of all respiratory and enteric pathogens. The Mixed Methods Appraisal Tool (MMAT) for quantitative descriptive studies was used to assess the risk of bias for eligible studies. Results of the MMAT assessment can be found in the supplemental material Supplementary file 5 (Appendix D).

### Analysis

We conducted preliminary data cleaning and qualitative coding of free text fields in Excel. Age categories were coded as: young children (< 5 years or before school entry), school-aged children under 18 years of age, adults age 18 or older, all ages, or other age groups. The categories for source population were decided based on common themes in included studies: general population, work/school setting (if the eligibility criteria or recruitment mechanism indicated only individuals within a certain institution were included), recruitment from a previously existing study cohort, or symptomatic cases. Studies were coded as retrospective, meaning that participants provided data on contacts that occurred in the past during a specified recall period, or prospective, meaning that participants were directed to record contacts as they occurred over a specified timeframe. Reporting periods were binned based on the frequency observed in the data. Duration and distance of interactions were standardized to meters or minutes/seconds (e.g. “2 meters”, “6 feet”, and “2 arms lengths” were standardized to “2 meters”). We conducted the final data cleaning and data analysis using R version 4.4.1.

## Results

Our search strategy identified 3438 unique articles (Ovid Medline = 1224, Embase = 1480, Scopus = 2905, Global Index Medicus = 58, 2227 duplicates removed [2147 by EndNote, 80 by Covidence], 2 retractions removed). Title and abstract screening identified 3243 irrelevant articles (e.g. contact allergies, fluid mechanics, contact lenses, soil/metal mixing, linguistics), and, after full-text review for eligibility, 112 studies were included Supplementary file 2 (Appendix A). A full catalogue of contact definitions used by included studies is available in the Supplementary material 4 (Appendix C).

### Study designs and settings

**Fig. 1:**
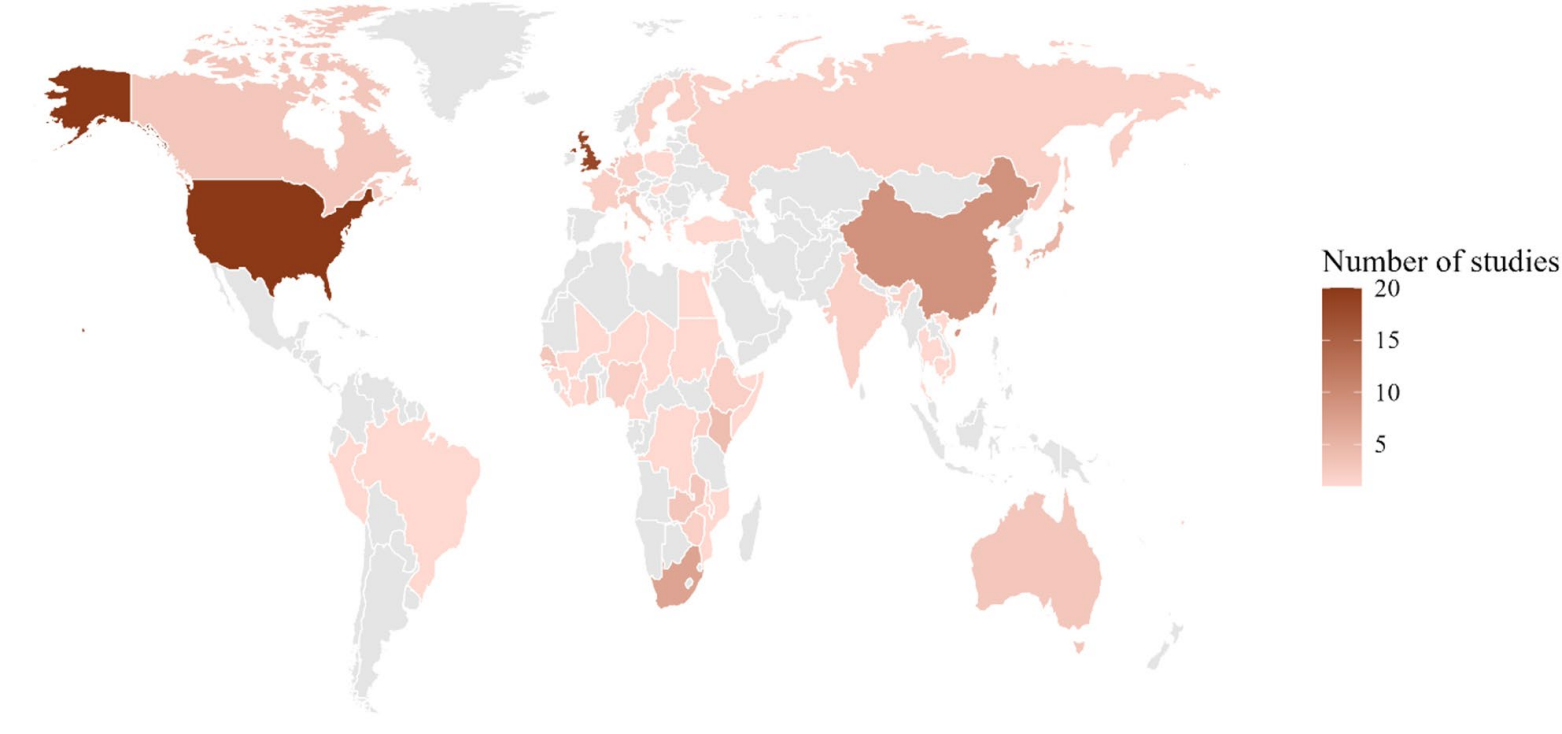
Global heatmap of contact study data collection (2005–2024). Studies that were conducted in multiple countries are represented once per country in which they were conducted

Most studies (*n* = 76, 68%) had a retrospective design. Overall, half of studies had no age restriction for their participants (*n* = 56), 29% (*n* = 32) enrolled only adults, 12% (*n* = 13) enrolled school-aged children only, and 3.6% (*n* = 4) enrolled only young children/infants (Table 1). Studies with a prospective design more often included only adults and older age groups compared to retrospective designs. The median sample size was 1,268 participants, with a minimum of 30 (Oh et al. 2020 and Oh et al. 2021) and a maximum of 10.7 million (Taube et al. 2024). Forty-one studies (37%) explicitly adapted their instrument from POLYMOD. Paper surveys were more common among prospective designs (*n* = 17, 47%) than retrospective designs (*n* = 4, 5.3%) which preferentially used electronic survey platforms (*n* = 31, 41%). Few studies longitudinally followed participants over multiple surveys (*n* = 13, 12%), and a third of studies (*n* = 36, 32%) used convenience sampling to recruit participants. The plurality of studies was conducted (in part or entirely) either in the United States (*n* = 20, 18%) or the United Kingdom (*n* = 18, 16%), followed by China (not including Taiwan or Hong Kong; *n* = 9, 8.0%) and South Africa (*n* = 7, 6.2%). Only three studies were conducted in the WHO Eastern Mediterranean Region (Fig. 1). The main limitations of study quality as assessed by the MMAT were representativeness and risk of non-response bias, primarily due to the sampling method used Supplementary material 4 (Appendix C). For example, response rates to survey invitations were typically low, or source populations were poorly-enumerated (e.g. invites via social media).

**Table 1:** Characteristics and design of social mixing studies (2005–2024)

Characteristic	Overall *N* = 112	Prospective *N* = 36	Retrospective *N* = 76
Age group^*1*^			
All	56 (50%)	19 (53%)	37 (49%)
Young children	4 (3.6%)	2 (5.6%)	2 (2.6%)
School-aged children	13 (12%)	7 (19%)	6 (7.9%)
Adults	32 (29%)	8 (22%)	24 (32%)
Other	7 (6.3%)	0 (0%)	7 (9.2%)
Source population			
General population	76 (68%)	23 (64%)	53 (70%)
Work/school setting	27 (24%)	12 (33%)	15 (20%)
Existing study cohort	7 (6.3%)	0 (0%)	7 (9.2%)
Symptomatic cases	2 (1.8%)	1 (2.8%)	1 (1.3%)
Sample size, median (IQR)	1268 (431, 3176)	564 (144, 1351)	1818 (595, 4205)
Study design			
Single survey	82 (73%)	26 (72%)	56 (74%)
Repeated cross-sectional	17 (15%)	5 (14%)	12 (16%)
Longitudinal	13 (12%)	5 (14%)	8 (11%)
Adapted from POLYMOD survey	41 (37%)	19 (53%)	22 (29%)
Sampling method			
Stratified random	49 (44%)	15 (42%)	34 (45%)
Convenience	36 (32%)	12 (33%)	24 (32%)
Random	18 (16%)	5 (14%)	13 (17%)
Unspecified	5 (4.5%)	3 (8.3%)	2 (2.6%)
Snowball	3 (2.7%)	1 (2.8%)	2 (2.6%)
Census	1 (0.9%)	0 (0%)	1 (1.3%)
Survey platform			
Paper survey	21 (19%)	17 (47%)	4 (5.3%)
Electronic survey	36 (32%)	5 (14%)	31 (41%)
In person interview	17 (15%)	1 (2.8%)	16 (21%)
Phone interview	10 (8.9%)	0 (0%)	10 (13%)
Multiple methods	12 (11%)	4 (11%)	8 (11%)
Unspecified	16 (14%)	9 (25%)	7 (9.2%)
Respondent			
Self	67 (60%)	22 (61%)	45 (59%)
Parent/guardian	4 (3.6%)	1 (2.8%)	3 (3.9%)
Self or parent/guardian	31 (28%)	11 (31%)	20 (26%)
Self or other shadow^*2*^	10 (8.9%)	2 (5.6%)	8 (11%)
Length of study (months)	3 (1, 7)	2 (1, 7)	3 (1, 7)
Unknown	2	1	1

^1^Young children include infants or toddlers before the age of school entry. School-aged children are those that are or can be enrolled in pre-K through age 17. Adults include participants over the age of 18, including university students. “Other” includes young children and adults (n = 1), any minor children under 18 (n = 1), and school-aged children and adults (n = 5)

^*2*^Other shadows include household members, teachers, or other individuals identified by the participant as someone with knowledge of the social contact activities of the participant during the survey period

**Fig. 2:**
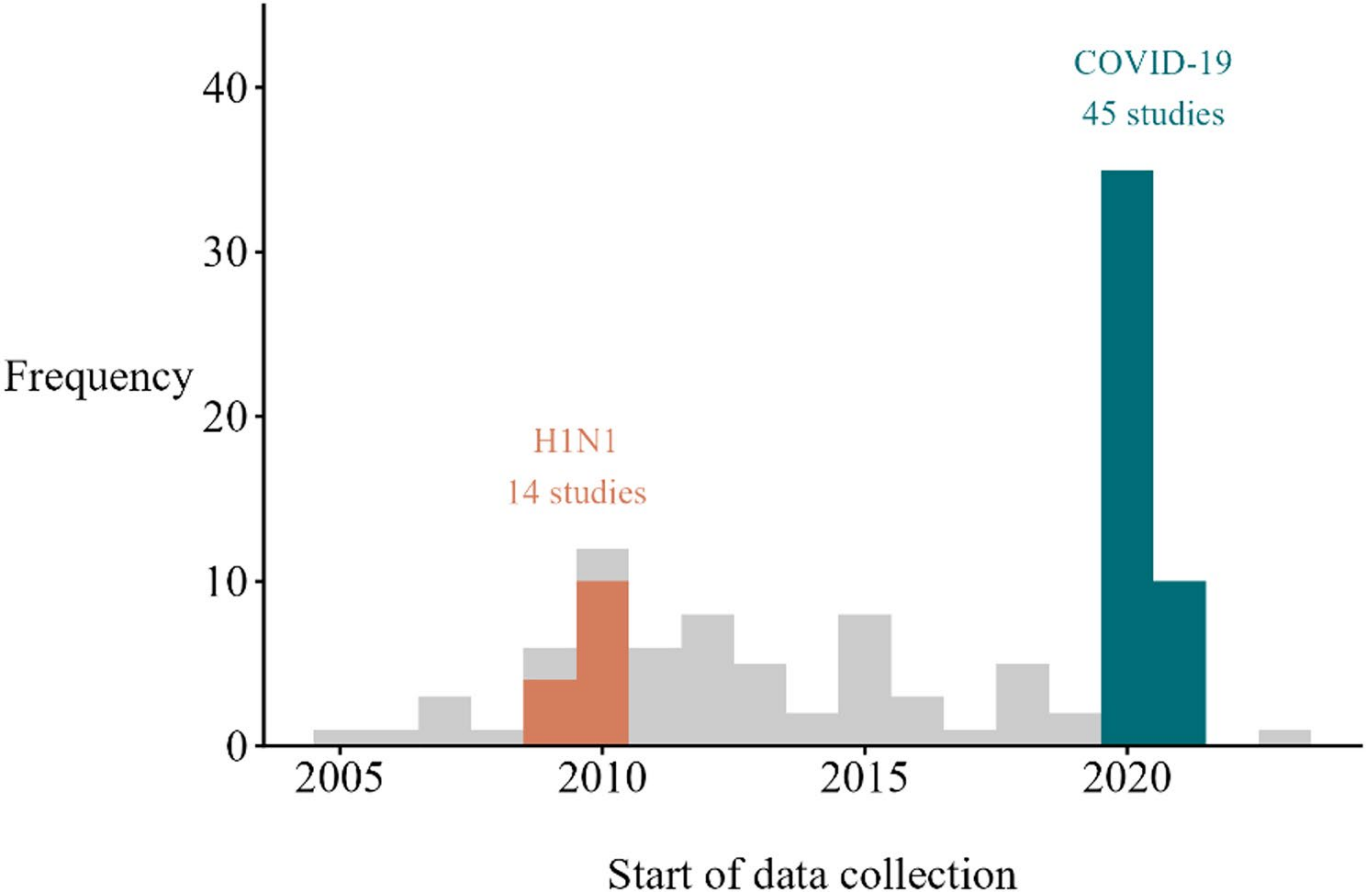
Histogram of the start of data collection for social mixing surveys from 2005 to 2024. The H1N1 pandemic is defined as the time period from June 2009 to July 2010, and the COVID-19 pandemic is defined as the time period from March 2020 to December 2021. Two studies that did not report the month and year of data collection are not included

**Fig. 3:**
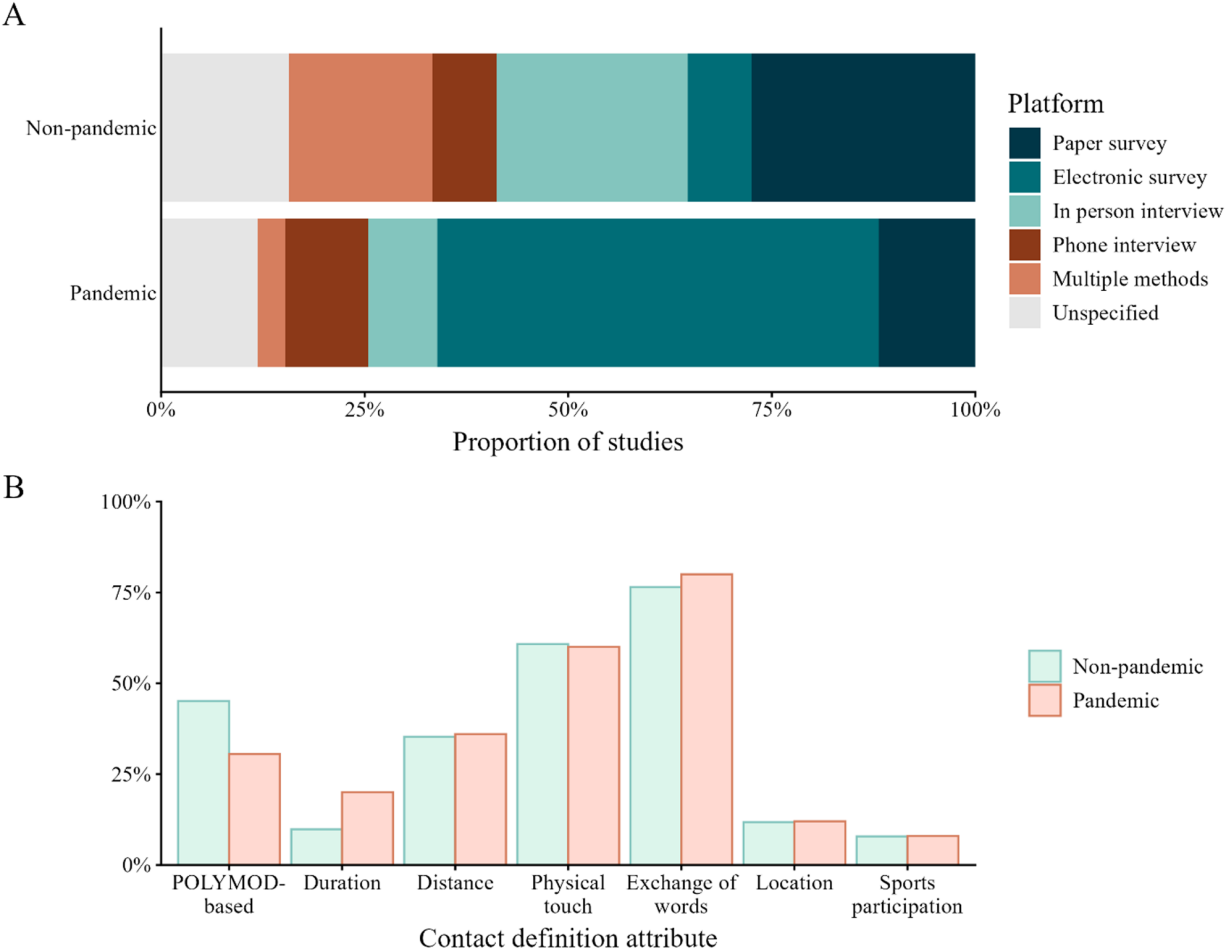
Comparison of survey design (**A**) and contact definition elements (**B**) during and outside pandemic periods (H1N1: June 2009-August 2010, COVID-19: March 2020-December 2021) from social mixing studies conducted between 2005 and 2024. POLYMOD-based definitions include studies that used this definition exactly (“either skin-to-skin contact such as a kiss or handshake [a physical contact], or a two-way conversation with three or more words in the physical presence of another person but no skin-to-skin contact [a nonphysical contact]”) or adapted the definition to a local context. Panel B categories on the horizontal axis are not mutually exclusive, so a single study may be represented more than once (e.g. POLYMOD-based definitions also included exchange of words and physical touch)

The frequency of new social mixing studies had two temporal peaks: first during the H1N1 pandemic (14 social mixing studies initiating data collection), and second during the COVID-19 pandemic (45 studies). Two studies did not report the start of their data collection period (Fig. 2). Half (*n* = 7, 50%) of studies initiated during the H1N1 pandemic enrolled participants of all ages, and a third (*n* = 4, 29%) enrolled only school-aged children. Studies that started during the COVID-19 pandemic were more likely to enroll only adults (*n* = 21, 47%) compared to those started at other times (*n* = 10, 15%). Electronic surveys were substantially more common during pandemic periods compared to other times (*n* = 32, 54% vs. *n* = 4, 8%, respectively; Fig. 3). Non-pandemic studies often used paper surveys (*n* = 14, 27%) and in-person interviews (*n* = 12, 24%) to collect data. Studies conducted during pandemic periods were also more likely to use retrospective designs (*n* = 46, 78%) compared to non-pandemic studies (*n* = 29, 57%).

### Contact definitions

The most common reporting period was one day (*n* = 73, 65%), and few required participants to record contact data for longer than 1 week (*n* = 2, 1.8%, Table 2). A minority of studies distinguished between contacts that occurred indoors vs. outdoors (*n* = 17, 16%), and 28% (*n* = 30) allowed participants to record contacts with large groups separately from individually named contacts. Collection of large group contacts was more common in retrospective surveys (*n* = 28, 38%) than prospective surveys (*n* = 2, 5.7%). Most studies asked participants to characterize contacts as household or extra-household (*n* = 78, 70%), but studies were inconsistent whether this identified household members or contacts that occurred within the home. Most studies separately collected the number of household members as baseline characteristic. Over half (*n* = 65, 59%) asked participants whether their contacts involved physical touch.

The contact definitions used in the study instruments were almost always available in the published material (*n* = 105, 93%). Relevant contacts were most often defined either in whole or in part by an exchange of words (*n* = 79, 77%) or physical touch (*n* = 61, 59%) between parties, with prospective designs more often using physical touch to define contacts than retrospective studies (79% of prospective studies [*n* = 27] and 49% of retrospective studies [*n* = 34]). Retrospective studies far more frequently used the duration of the interaction to define relevant contacts than prospective studies (*n* = 15, 22% vs. *n* = 1, 2.9%), but the duration of interest was variable, ranging from ≥ 5 s (Andrejko et al. 2022) to ≥ 15 min (Gravagna et al. 2022 and Powers et al. 2022). Studies initiated during pandemic periods were less likely to use or adapt the POLYMOD instrument (*n* = 18, 31%) than non-pandemic studies (*n* = 23, 45%), but more likely to define relevant contacts by interaction duration (*n* = 10, 17% vs. *n* = 5, 10%; Fig. 3), typically specifying interactions that lasted more than a few minutes. Among studies that specified a distance between parties (*n* = 37), 2 m was the most referenced distance (*n* = 23, 62%).

**Table 2:** Characteristics of contact definitions used by social mixing studies (2005–2024)

Characteristic	Overall *N* = 112	Prospective *N* = 36	Retrospective *N* = 76
Reporting period			
<1 day	3 (2.7%)	2 (5.6%)	1 (1.3%)
1 day	73 (65%)	23 (64%)	50 (66%)
2 days − 1 week	14 (13%)	7 (19%)	7 (9.2%)
>1 week	2 (1.8%)	1 (2.8%)	1 (1.3%)
Other^*1*^	13 (12%)	0 (0%)	13 (17%)
Unspecified	7 (6.3%)	3 (8.3%)	4 (5.3%)
Contact data collected			
Duration of contact collected	66 (59%)	28 (78%)	38 (51%)
Unknown	1	0	1
Symptom status of participant collected	24 (21%)	5 (14%)	19 (25%)
Large group contacts (vs. named contacts)	30 (28%)	2 (5.7%)	28 (38%)
Unknown	3	1	2
Outdoor contacts (vs. indoor contacts)	17 (16%)	4 (11%)	13 (18%)
Unknown	4	0	4
Household contacts (vs. extra-household contacts)^*2*^	78 (70%)	22 (61%)	56 (75%)
Unknown	1	0	1
Physical touch (vs. close proximity)	65 (59%)	29 (81%)	36 (49%)
Unknown	2	0	2
Terms by which relevant contacts were defined			
Exchange of words	79 (77%)	30 (88%)	49 (71%)
Physical touch	61 (59%)	27 (79%)	34 (49%)
Distance between parties	37 (36%)	12 (35%)	25 (36%)
Duration of interaction	16 (16%)	1 (2.9%)	15 (22%)
Location of interaction	14 (14%)	4 (12%)	10 (14%)
Sports participation	8 (7.8%)	1 (2.9%)	7 (10%)

^1^Other recall periods include prompts like “a typical day” or “your most recent shift”

^*2*^The term “household contacts” represents the usage of the term in the source material. Some studies defined household contacts as contacts that occurred between household members, regardless of location, and others defined household contacts that occurred within the home either with other household members or with visitors to the home

### Three-pathogen framework

Based on our review of transmission biology literature, we determined that contact distance or exchange of words (in this context a proxy for both distance between participant and contact and production of large aerosols) defined the relevant contact parameters for influenza. There is some evidence that influenza may be transmitted by smaller aerosolized particles, as well, and indirect contact may be a prevalent yet uncommon route of transmission, although we determined that the impact on contact rate estimates by excluding this route would likely be negligible. Because tuberculosis is transmitted by small particles with a long travel distance and short viability outside of the host, we concluded that household contacts or other contacts of long cumulative duration, indoor contacts in settings with poor ventilation, and large group contacts were pertinent for transmission. These criteria align with current WHO guidelines on screening of household and other close contacts with whom index cases have sustained interaction [20] as well as epidemiologic evidence of substantial community transmission in congregate settings with poor ventilation [21]. Finally, because norovirus is transmitted via direct or indirect contact and can persist on surfaces, physical touch contacts and household contacts that share living spaces such as kitchens and lavatories comprise the set of at-risk contacts. Eighty-six studies (77%) included in this review defined contacts using parameters that correspond to transmission of influenza by direct deposition, mostly using retrospective designs (*n* = 56, 65%) and random or stratified random sampling (*n* = 52, 60%). Seven studies (6.2%) captured information that corresponds to airborne transmission of tuberculosis, all using retrospective designs and six of seven using random or stratified random sampling, and half (*n* = 56) of studies collected such data for norovirus contacts (*n* = 38, 68% retrospective; *n* = 41, 75% random or stratified random; Table 3).

**Table 3:** Transmission biology, environmental persistence, and contact parameters of influenza, tuberculosis, and norovirus

	Influenza	Tuberculosis	Norovirus
Transmission route [19]	Direct deposition (primary transmission route) [22], airborne [23]	Airborne [24]	Direct and indirect contact (fecal-oral) [25]
Host entry point	Upper respiratory tract (eyes, nose, mouth) [22]	Lower respiratory tract, terminal bronchi and alveoli [24]	Gastrointestinal tract (mouth, nose)
Viability outside of host	Up to 2 h in smaller droplets [23, 26]	Up to 1 h [27]	2 months or more in water, approx. 3 days on surfaces [28]
Relevant particle size (diameter)	Most commonly large (approx. 5–100 μm), but small particles (up to approx. 5 μm) may be infectious [22, 23, 26]	Small (up to approx. 5 μm) [24, 29]	Usually direct/indirect contact only, but small-particle aerosolized vomitus may be infectious [25]
Particle travel distance	Up to 2 m [23]	> 2 m [30]	For aerosolized vomitus, possibly a few meters [31, 32]
Minimal infectious dose (ID_50_)^†^	0.7–3.5 plaque-forming units (PFU) for airborne particles, 89–224 PFU for direct deposition in eyes, nose, or mouth [33]	< 10 colony-forming units, [34, 35] highly dependent on host factors*	18-2800 genomic equivalents, dependent on host factors^‡^ [25]
Relevant contact parameters	Distance or exchange of words	Large groups, indoor contacts, and household contacts or contacts of long duration	Household contacts and contacts involving physical touch
Number of studies that include relevant parameters	86 (77%)	7 (6.2%)	56 (50%)

^†^ID_50_ is the infectious dose needed to produce sustained infection in 50% of hosts, usually established in animal models

*Host factors leading to a higher risk of tuberculosis infection include immunocompromising conditions such as autoimmune disease, organ transplant, and cancers

^‡^Host factors influencing norovirus infection include secretor status, in which those with an inactive FUT2 gene (non-secretor phenotype) have protection against infection by most norovirus genotypes

## Discussion

We evaluated 112 social mixing studies that initiated data collection in or after 2005. While new social mixing studies were conducted every year between 2005 and 2021, pandemics sparked waves of new studies as the importance of social mixing in understanding the spread of infectious disease became more widely recognized. Despite this, several regions still largely lack empirical social mixing data, limiting global generalizability. In fact, countries with the highest communicable disease burden such as sub-Saharan Africa, South and Southeast Asia, and South America [36] were less likely to be represented in social mixing literature. Social mixing patterns are heterogenous between and within regions due to cultural and economic factors such as urban or rural geography, economy, socioeconomic status, schooling, and cohabitation norms. Social mixing studies predominantly conducted in high- and upper middle-income countries with low infectious disease burden may misrepresent mixing patterns when projected to low-income countries with higher disease burden [14]. 

In addition, the increased importance of, and interest in, social mixing patterns during the COVID-19 and H1N1 pandemics resulted in over half of studies included in this review being conducted during periods where contact behaviors were altered or substantially disrupted, and likely non-representative of non-pandemic periods. Studies conducted during pandemic periods more often used retrospective designs that can suffer from biases in which casual contacts are systematically underreported. While casual contacts were substantially reduced during COVID-19 social distancing restrictions, potentially limiting these biases, contact rate estimates are likely substantially lower than they would be during non-pandemic periods, particularly among community contacts. Pandemic period estimates are best suited for modeling epidemic scenarios in which similar public health measures are expected to be implemented, and, if estimates must be used outside of these contexts, modelers should note this as a limitation.

Almost a third of studies used convenience sampling to create their study populations, which can introduce selection bias and lack of representativeness because, with a poorly enumerated source population, sampling weights are difficult to define. Few studies focused on young or school-aged children < 18 years old, who play important roles in the spread of respiratory and enteric infections [25]. A majority used cross-sectional, single-day designs that do not allow for evaluation of recurring vs. new contacts, the distribution of which provides insight into cumulative risk of transmission to frequent contacts and the ability of an infection to spread quickly outward from individuals with a high proportion of new contacts. In addition, retrospective designs were more common despite their susceptibility to information bias. The more recent popularity of electronic surveys make prospective designs more feasible, for example through short, frequent surveys delivered via smartphone apps, but electronic surveys were more common in this review among retrospective designs.

Critical modifiers of transmission risk for our archetypal pathogens – influenza, tuberculosis, and norovirus – were under-measured. For example, because influenza is primarily transmitted via direct deposition into the upper airways, shorter distance close contacts are most relevant. However, a quarter of studies identified for this review did not use definitions or collect data that align with these close contacts. Because tuberculosis can be carried by small aerosols, transmission occurs though sustained shared-air exposures either within the household or other shared spaces, including contacts not identified through individual reporting. Most studies in this review did not collect sufficient information to identify all contacts relevant to tuberculosis transmission. Collecting large group contacts or stratifying indoor or outdoor contacts was uncommon; ultimately we found that only seven studies in this review collected contact data aligned with airborne transmission of tuberculosis. Importantly, contacts with household members and contacts within the home were often conflated. While tuberculosis can be transmitted to visitors of the home, household members are at particularly high risk due to their extended exposure to potentially infectious aerosols, and household transmission is epidemiologically relevant to quantify. Finally, though most social mixing studies are designed to capture contacts relevant for transmission through the air, these data are also often used to model person-to-person transmission of enteric pathogens [4, 37, 38]. Aerosolization of particles from the respiratory tract through breathing or talking are unlikely to be interactions with transmission potential and should not be included in the calculation of a contact rate, so enumerating household contacts (in particular, household contacts that are likely to have shared food or lavatory facilities), and touch contacts is crucial in modeling efforts. Half of studies in this review met these criteria. Norovirus, as well as other common enteropathogens, is also commonly transmitted by indirect contact or reservoirs such as food or water, and therefore relevant contacts may be misrepresented by social mixing studies requiring face-to-face interactions.

To allow more flexible use of social mixing data that may be used in a variety of transmission contexts, we recommend first clearly defining a target pathogen profile and aligning the quantified interactions to the relevant transmission route(s). Social mixing surveys should collect whether the contact occurred indoors and whether the contact was a member of the participant’s household. In addition, to capture contacts at risk for large-scale airborne transmission events and prevent bias from differential recall of high intensity contacts, surveys should collect large group contacts separately from individual contact events. Those seeking to parameterize models with social mixing data should critically evaluate the study design, setting, and contacts collected for likelihood of bias and expected direction, as well as alignment with the transmission route of interest (for example, models of outbreaks of respiratory viruses spread via droplets in schools can identify social mixing studies conducted among school-aged children that identify close contact interactions). Examples of contact data considerations for specific research questions are presented in Table 4. While we considered three archetypal pathogens as the framework for this this analysis, we recognize that adjustments may be necessary to define contacts relevant for other pathogens. For example, all pathogens which are airborne are not similarly infectious (e.g. measles is far more infectious than tuberculosis) or are transmitted by multiple routes (enteroviruses such as cholera may also be transmitted by contaminated water). As we have demonstrated for our three pathogens of interest, modeling efforts focused on other pathogens should explicitly consider the route of transmission in the choice of contact rate.

This review provides a comprehensive catalog of social contact definitions across respiratory and enteric contexts from studies published from 2005 to 2024. We used rigorous review and data collection methods, requiring consensus on all eligibility and data elements. However, this review also has limitations. First, we included only studies published prior to the query date in August 2024, but there may be additional studies meeting eligibility criteria that had initiated data collection but were yet to be published. In addition, studies were inconsistent in their definitions of household contacts, which should be considered particularly for questions in which new vs. recurrent contacts are important and in evaluation of contact tracing interventions in which household members are identified separately. Finally, transmission events in observational research are never directly observed, so social mixing studies are at best approximations of potential transmission events based on what is known about the biology of the pathogen and investigations which have inferred linkages between the nature of pathogen exposure to discrete transmission events. While our recommendations represent an evidence-based data collection paradigm, the direct relationship between the observed contacts and unobserved transmission events will always be imperfect. Nevertheless, data from social mixing surveys remain central to parameterizing models for transmission, and integrating setting, duration/distance, household status, and shared space to align with pathogen-specific transmission routes may yield more accurate inference from transmission models. Future analyses should be conducted to validate models using different contact types against empirical data to quantitatively evaluate the impact of conceptual misalignment with model estimates.

**Table 4:** Example considerations for use of social mixing data in models of infectious disease transmission

Research question	Considerations for use of social mixing data
How does an intervention targeting household contacts reduce overall tuberculosis incidence in South Africa?	• Are the data representative low-/middle-income countries? • Can household contacts be distinguished and are contacts with household members or that occur within the home of greater importance? • Consider if community contacts are of interest and whether data adequately represent risk (indoor/outdoor location, duration) and limit underreporting with prospective design.
What is the impact of age-targeting vaccination for norovirus?	• Does the study include data from young children who may be targets for norovirus vaccination? • Does the sampling method allow for estimation of representative mixing across age groups? • Should contact patterns be distinguished between symptomatic and non-symptomatic periods, and is this discernable in the data?
How might social distancing measures in schools mitigate flu transmission and burden in the school setting?	• Consider if data collected during pandemic periods may be of interest for benchmarking socially distanced contact rates. • Identify studies conducted within school settings for context-specific mixing patterns. • (Similar to above) should contact patterns be distinguished between symptomatic and non-symptomatic periods, and is this discernable in the data?

## Conclusions

We synthesized a large amount of information from studies conducted in a variety of contexts to inform the design of future social mixing studies and use of social mixing data in models. Critical modifiers of transmission risk such as location, household membership, and shared space with large numbers of people were under-measured. For modeling efforts, we recommend choosing contact parameters that align with the research question and transmission route being modeled that have been compiled and made available as a part of this review, and for social mixing studies, aligning contacts and data elements to the target pathogen(s) of interest.

## Supplementary Information

Below is the link to the electronic supplementary material.

Supplementary Material 1: Review Data

Supplementary Material 3: Appendix B

Supplementary Material 2: Appendix A

Supplementary Material 4: Appendix C

Supplementary Material 5: Appendix D

## Data Availability

The data generated and analyzed in this review are available in the supplementary material.
