# Supplementary material for: Relevance of social contact definitions for use in infectious disease transmission modeling: a systematic review and recommendations
Source: BMC Infect Dis. 2026 Mar 18;26:836. doi: 10.1186/s12879-026-12938-y (PMC13112660; doi:10.1186/s12879-026-12938-y)
Supplement: Supplementary file 2 — Supplementary Material 3: Appendix B [file 12879_2026_12938_MOESM2_ESM.docx]

**Appendix B**

**Data Extraction Template**

### Article Identification

| **Study ID** |  |
| --- | --- |
| **Title**  Title of paper / abstract / report that data are extracted from |  |
| **Lead author** |  |
| **Article type** | - Pre-print - Research article |

### Methods

| **Inclusion criteria**  Enter each criterion verbatim, separated by a semi-colon “;” |  |
| --- | --- |
| **Exclusion criteria**  Enter each criterion verbatim, separated by a semi-colon “;” |  |
| **Country in which the study was conducted** | - United States - UK - Canada - Multiple - Other _______ |
| **Sample size**  Enter numeric only |  |
| **Timeframe of data collection**  Enter date or date range in month and year (e.g. January 2013-January 2015”, “March 2021”) |  |
| **Study design**  “Repeated cross-sectional” refers to studies that resample from the source population at each time point. “Longitudinal” are surveying the same participants multiple times. | - Single retrospective survey - Prospective diary - Repeated cross-sectional - Longitudinal |
| **Sampling method**  As described in the method section. Census means that the entire source population was included in the study. | - Random - Stratified random - Census - Convenience - Snowball - Unspecified - Other _________ |
| **Verbatim contact definition**  Pull the contact definition exactly as stated by the authors. |  |
| **Contact definition elements**  Enter “Y” or “N” if the element was a required criterion for determining contact, or an optional criterion. For duration, distance, and location, enter the specified value. | \|  \| Required \| Optional \| Value \| \| --- \| --- \| --- \| --- \| \| Duration \|  \|  \|  \| \| Distance \|  \|  \|  \| \| Location \|  \|  \|  \| \| Exchange of words \|  \|  \|  \| \| Sports \|  \|  \|  \| \| Physical touch \|  \|  \|  \| |
| **Where was the contact definition located in the paper?** | - Main body - Appendix/supplement - Unspecified contact definition |
| **Is the study based off of POLYMOD** | - Yes - No |
| **Relation of participant to the person answering the survey**  The participant refers to the person about whom the contact data pertains, i.e. the “ego”. | - Self - Parent/guardian - Self and/or parent/guardian - Other _______ |
| **Data collection platform** | - Electronic survey - Paper survey - In-person interview - Phone interview - Multiple methods - Unspecified |
| **Recall period**  Enter the time period (relative to the survey time) that the participant is answering questions about. |  |
| **Are large group contacts collected separately from individual contacts?** | - Yes - No - Unknown |
| **Maximum number of individual contacts allowed to be reported**  If there are separate maximums for different types of individual contacts, sum across types of contacts |  |
| **Do they differentiate between indoor/outdoor contacts?** | - Yes - No - Unknown |
| **Do they differentiate between household/non-household contacts?** | - Yes - No - Unknown |
| **Do they collect the duration of the contact?** | - Yes - No - Unknown |
| **Do they differentiate between physical touch/non-physical touch contacts?** | - Yes - No - Unknown |
| **Do they collect participant symptom status?** | - Yes - No - Unknown |

### Results

| **Contact rate measure**  e.g. mean (SD), median (IQR), mean (95% CI) |  |
| --- | --- |
| **Contact rate estimate**  In the format as above. If multiple, separate each result and description by a semi-colon “;” |  |
