# Supplementary material for: Relevance of social contact definitions for use in infectious disease transmission modeling: a systematic review and recommendations
Source: BMC Infect Dis. 2026 Mar 18;26:836. doi: 10.1186/s12879-026-12938-y (PMC13112660; doi:10.1186/s12879-026-12938-y)
Supplement: Supplementary file 3 — Supplementary Material 2: Appendix A [file 12879_2026_12938_MOESM3_ESM.docx]

**Appendix A**

**Search Strategy and Results**

**Searches executed**: 8/20/2024

**Total search results**: 5667

Total duplicates removed in Endnote: 2147

Retractions removed in Endnote: 2

### Ovid Medline (1224)

| **#** | **Search Statement** | **Results** |
| --- | --- | --- |
| 1 | ((contact adj2 (rate* or study or studies or pattern* or histor* or network*)) or (mixing adj2 (study or studies or pattern* or social* or behavio?r*))).ti,ab,kw,kf. | 10652 |
| 2 | exp “Surveys and Questionnaires”/ | 1258992 |
| 3 | (survey* or questionnaire* or diary or diaries).ti,ab,kw,kf | 1491402 |
| 4 | 2 or 3 | 2196553 |
| 5 | 1 and 4 | 1489 |
| 6 | Limit 5 to yr=”2005-Current” | 1224 |

### Embase (1480)

((contact NEAR/2 (rate* OR study OR studies OR pattern* OR histor* OR network*)) OR (mixing NEAR/2 (study OR studies OR pattern* OR social OR behavio$r*))) AND (‘questionnaire’/exp OR survey* OR questionnaire* OR diary OR diaries) AND [2005-2024]/py

### Scopus (2905)

( TITLE-ABS-KEY ( ( contact W/2 ( rate* OR study OR studies OR pattern* OR histor* OR network* ) ) OR ( mixing W/2 ( study OR studies OR pattern* OR social* OR behavio*r* ) ) ) AND ( INDEXTERMS ( “Surveys and Questionnaires” OR questionnaire ) OR TITLE-ABS-KEY ( survey* OR questionnaire* OR diary OR diaries ) ) ) AND PUBYEAR > 2004 AND PUBYEAR < 2026

### Global Index Medicus (58)

(“contact rate” OR “contact rates” OR “contact study” OR “contact studies” OR "contact patterns” OR “contact history” OR “contact histories” OR "contact network” OR "contact networks” OR “mixing study” OR “mixing studies” OR “mixing pattern” OR “mixing patterns” OR “mixing behavior” OR “mixing behaviors” OR “mixing behaviour” OR “mixing behaviours” OR “social mixing”) AND (survey* OR questionnaire* OR diary OR diaries


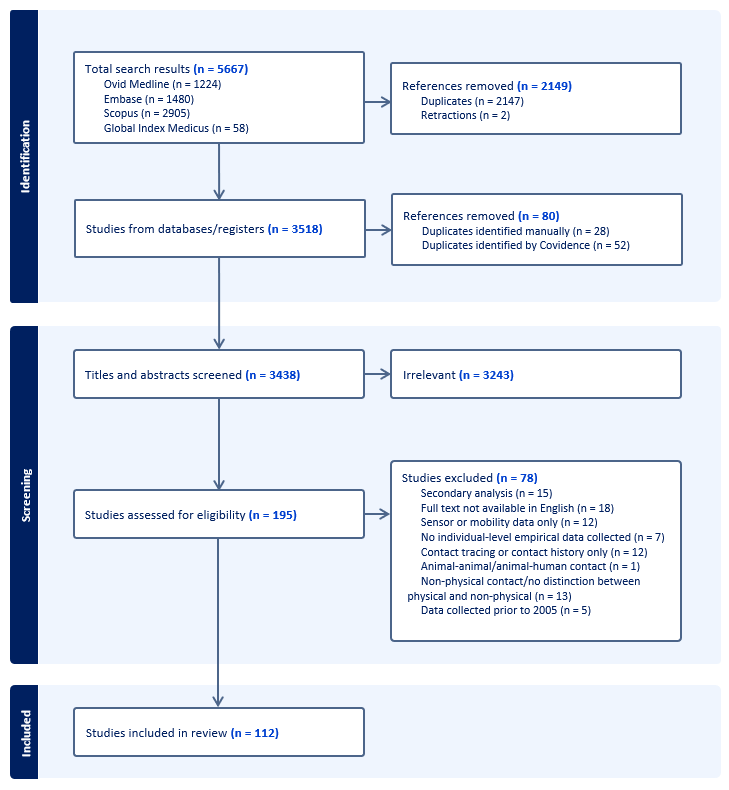


**Figure S1**. PRISMA diagram of inclusion of social mixing studies from 2005-2024
