# Supplementary material for: Relevance of social contact definitions for use in infectious disease transmission modeling: a systematic review and recommendations
Source: BMC Infect Dis. 2026 Mar 18;26:836. doi: 10.1186/s12879-026-12938-y (PMC13112660; doi:10.1186/s12879-026-12938-y)
Supplement: Supplementary file 4 — Supplementary Material 4: Appendix C [file 12879_2026_12938_MOESM4_ESM.docx]

**Appendix C**

**Catalogue of Contact Definitions and Contact Rates**

**Table S1**. List of contact definitions and estimated contact rates by social mixing studies (2005-2024)

| **Study** | **Setting** | **Contact definition** | **Contact rate*^1^*** | **Pathogen alignment** |
| --- | --- | --- | --- | --- |
| Dodd 2016 | Multiple | Close contact: contact with someone with whom the interviewee had a face-to-face conversation that was longer than a greeting and within an arm’s reach; casual contact: contacts with people who were inside buildings other than the interviewee’s home that the interviewee had visited | Close contact rate of adult: mean (95% CI) 4.9 (4.6, 5.2) Casual contact rate of adult: 10.4 (9.3, 11.6) | influenza |
| English 2018 | Canada | Direct patient contact: Two or more individuals coming within 1 m (approximately 3 ft) of each other for 2 min or more. Indirect contact: Two or more individuals co-locating in the same room but not closer than 1 m. |  | influenza |
| lePolaindeWaroux 2018 | Uganda | Two-way conversational encounters lasting for ≥5 min | Mean 7.2, median 7 (range 0-25) | influenza, norovirus |
| McCreesh 2016 | Multiple | People in buildings (other than their own home) that they had entered |  |  |
| Pilny 2021 | United States | Being in the same proximity within six feet with anybody for at least a duration of 10 min | baseline mean (SD) 9.28 (8.69); retrieval cue 10.61 (7.68); context-approach 13.13 (7.60) | influenza |
| Stefkovics 2024 | Hungary | Only face-to-face interactions lasting a few minutes or longer were recorded, excluding shorter encounters (e.g. greetings) and interactions over the Internet or telephone. |  | norovirus |
| Ajelli 2017 | Russia | A two-way conversation of at least five words in the physical presence of another person | Mean 9.8 excluding additional professional contacts; 15.2 including professional contacts. | influenza |
| Andrejko 2022 | United States | Interaction within 6 feet with a non-household member lasting over 5 s | first wave mean (95% CI) 2.28 (1.99, 2.58); second wave 3.24 (2.93, 3.58); third wave 3.31 (95% CI: 3.01, 3.62) | NA, NA |
| Auranen 2021 | Finland | A two-way conversation with at least a few words exchanged in presence of another person | adults mean (SD) 2.4 (2.6); children 3.5 (2.0) | NA |
| Backer 2023 | Netherlands | A conversation of at least a few sentences and/or a physical contact | period 1 frail mean (95% CI) 14 (12—17); period 1 not frail 19 (16—21); period 2 frail 1 (17—25); period 2 not frail 6 (23—30) | influenza, norovirus |
| Backer 2024 | Netherlands | Unique persons with whom the participant talked face-to-face, touched, kissed or played sports | baseline mean (95% CI) 17.8 (17.0–18.5) | influenza, norovirus |
| Beale 2022 | UK | All settings where they spent time during a recent 24 h period |  |  |
| Beraud 2015 | France | Talking to someone within a distance of less than 2 meters, or skin-to-skin touching | Median 8 (IQR 5–14) | influenza, norovirus |
| Bosetti 2021 | France | Either a physical contact such as a kiss or handshake, or a close contact such as face-to-face conversation at less than 1 m distance | Mean 3.30; Median 2 (95% quantiles 0-16) | influenza, norovirus |
| Brankston 2021 | Canada | Anyone who was met in person and with whom a short conversation occurred, or anyone with whom the respondent had physical contact | Survey 1: mean (IQR) 2.21 (0, 3) Survey 2: 2.17 (0, 3) Survey 3: 4.76 (0, 4)  Survey 4: 3.89 (0, 3) | influenza, tuberculosis, norovirus |
| Bridgen 2022 | UK | Someone with whom the participant had a face-to-face conversation with, excluding members of their own household | Mean (95% CI) 2.9 (2.7-3.0) | influenza |
| Danon 2013 | UK | A contact was defined as a person with whom the participant had a face-to-face conversation (within 3 m) and/or skin-on-skin physical touch | individual: mean 8.28; individual + group 28.50 | influenza, norovirus |
| DelFava 2021 | Kenya | An interaction between two individuals, either physical (involving skin-to-skin contact), or non-physical (involving a two-way conversation with three or more words in the physical presence of another person, but no skin-to-skin contact) | Mean 8.92 (median 8, IQR 5–12) | influenza, norovirus |
| DeStefano 2011 | United States | For adults: Speaking interactions represent the total number of people with whom a survey respondent had a face-to-face conversation lasting >1 minute during the day before the interview. Close-proximity contacts are those within 6 feet for >15 minutes during the day before the interview. For children: Potential social contacts are calculated as the sum of the number of people the responding parent estimated were present at various locations or activities attended by the child, including household, school, daycare, and other specified locations. | Adults (Male, Female): Speaking interaction mean (SD) =11.7 (19.5), 12.5 (14.6) Close proximity contact=13.2 (25.4), 12.2 (15.6); Children (male, female): Daily potential social contacts=96.5 (100.0), 121.9 (119.3) | influenza |
| Dobreva 2022 | Multiple | Anyone with whom [the respondent] exchanged at least a few words and was close enough to not need to raise [their] voice or [who they] had direct physical contact with (including handshaking or other contact) | Survey 1: mean 9 (SD = 16) - 41 (56), median 4 (IQR = 8)-20 (41) ; Survey 2: mean 13 (15) - 50 (53), median 8 (11) - 33 (40) | influenza, norovirus |
| Drolet 2022 | Canada | Either physical (handshake, hug, kiss) or non-physical (two-way conversation in the physical presence of the person, at a distance equal or less than 2 m, irrespective of masking). | Pre-pandemic mean (95% CI): 7.8 (95% CI: 7.2–8.5) Spring 2020 : 3.1 (95% CI: 2.6–3.5) Summer 2020: 5.0 (95% CI: 4.3–5.8) Fall 2020: 4.1 (95% CI: 3.7–4.5) Holidays 2020–2021: 2.9 (95% CI: 2.7–3.1) January 2021: 3.5 (95% CI: 3.0–3.9) February 2021: 4.0 (95% CI: 3.3–4.6) | influenza, norovirus |
| Ferraro 2014 | Multiple | Attending a market, wedding, funeral, ceremony (e.g. circumcision), social club, bar or other such event in the last week; resident in household | Household geometric mean (95% CI) people per room: 2.17 (2.14–2.21) Mean number of social gatherings: 0.83 |  |
| Fu 2012 | Taiwan | Physical contacts and those nonphysical contacts with verbal communication made within 2 meters | Mean (SD) 12.5 (9.3) | influenza |
| Ghilotti 2019 | Sweden | Having a distance between you of less than a meter for more than a minute |  | influenza, norovirus |
| Goeyvaerts 2018 | Belgium | A two-way conversation at less than 3 m distance or a physical contact involving skin-to-skin touching (either with or without conversation) | Mean density  weekday; 0.93 weekend; 0.94 | influenza, norovirus |
| Grijalva 2015 | Peru | A conversation with another person that is physically present and no farther than 3 meters, or a physical contact involving skin-to-skin touching, e.g. a kiss or handshake (either with or without conversation) | Reported contacts: median = 12 (IQR 8–20) Physical (skin-to-skin) contacts : 8.5 (5–14) | influenza |
| Hidano 2022 | UK | Physical contact is any sort of skin-to-skin contact, while non-physical contact is a contact that involves exchanging a few words face-to-face with or without 1 m distancing | Physical contacts: mean 0.57 (95% CI 0.38–0.76), Non-physical contacts without distancing: 1.86 (1.17–2.56), Non-physical contacts with distancing: 3.64 (2.76–4.52) | influenza, norovirus |
| Higgins 2023 | Brazil | Talked face to face or had any physical contact (i.e. handshake, hugs, kisses, contact while doing sports) with one or more people at the same time |  | influenza, tuberculosis, norovirus |
| Hoang 2021 | Belgium | (1) Two-way conversations during which at least three words were spoken and (2) contacts that involved skin-to-skin touching | mean: 18.4 (SD 24.3), median: 12 (IQR 6-21) | influenza, norovirus |
| Horby 2011 | Vietnam | Either skin-to-skin contact (a physical contact), or a two-way conversation with three or more words in the physical presence of another person but no skin-to-skin contact (a nonphysical contact) | Mean 7.7 (SD 3.9) | influenza, norovirus |
| Huang 2020 | China | Conversation with three or more words or physical contacts including handshakes, hugs, kisses, and ball games | Mean 6.2 (SD 3.3); 16.7 (14.1) including supplementary professional contacts | influenza, norovirus |
| Ibuka 2016 | Japan | Face-to-face meetings with words exchanged within a distance of 2 m | mean 15.3 (95% CI 14.4 to 16.3) and a median of 12 | influenza |
| Johnstone-Robertson 2011 | South Africa | Close contacts were defined as those involving physical touch (type I) or those involving a 2-way conversation with 3 or more words in the physical presence of another person without physical touch (type II). Casual contacts (type III) were defined as those occurring in an in-door location but not satisfying the criteria for a close contact | Physical contacts: median 12 (IQR 7–18), Close contacts: 20 (13–29), Indoor contacts: 30 (12–54) | influenza, NA, norovirus |
| Kiti 2014 | Kenya | Someone with whom the participant had a direct physical encounter (a ‘contact’), and involved direct skin-to-skin touch such as embracing, kissing or shaking hands | Mean (95% CI) 17.7 (16.7–18.7) |  |
| Kleynhans 2021 | South Africa | A two-way conversation of at least three words at a distance not requiring voices being raised (<2 m between individuals), with or without physical contact, or where physical contact took place without conversation | Median 14 (IQR 9-33) | influenza, norovirus |
| Kumar 2018 | India | Having had a face-to-face conversation within 3 feet, which may or may not have included physical contact | Median 17 (IQR 12-25) | influenza, NA |
| Kurahashi 2023 | Japan | Visit to a room in household |  |  |
| Kwok 2014 | Hong Kong | Face-to-face conversation or skin-on-skin contact | Range 18.0-18.6 | influenza, norovirus |
| Latsuzbaia 2020 | Luxembourg | A face-to-face conversation with more than three words at a distance of less than two meters | During lockdown: mean 3.2 (95% CI 3.1–3.3), median 3.0 (IQR 1–4) After lockdown: mean 7.1 (IQR 3–9) | influenza |
| Leach 2023 | United States | ‘Interactions’ to improve recall, and participants were provided with the following examples, ‘having a conversation, making a purchase, or standing closer than 6 feet to another person for a few minutes.’ Outdoor contacts explicitly excluded passing someone on the street without stopping to talk |  | influenza, tuberculosis |
| Leung 2017 | Hong Kong | Either skin-to-skin touch such as a handshake (a physical contact) or a face-to-face conversation with three or more words in the physical presence of both the participant and the contact within two meters | Mean 6.93 (SD 0.19, 95% CI 6.56-7.32) | influenza, norovirus |
| Leung 2023 | Cambodia | Either a two-way conversation in the physical presence of another person or physical skin-to-skin contact | Median 22 (IQR 13-40) | influenza, norovirus |
| Liang 2023 | China | Either a two-way conversation involving three or more words in the physical presence of another person or direct physical contact (e.g., a handshake) | Wuhan baseline mean 14.6 (95% CI 13.3, 16.1), outbreak 2.0 (1.9, 2.1), post-pandemic 3.9 (3.0, 5.3); Shanghai baseline 18.9 (17.7, 20.1), outbreak 2.3 (2.0, 2.8), post-epidemic 2.8 (2.4, 3.1); Shenzhen baseline 7.9 (7.3, 8.7), outbreak 2.2 (2.1, 2.3), post-epidemic 3.7 (2.8, 4.6); Changsha baseline 9.5 (8.7, 10.4), outbreak 2.2 (2.1, 2.3); post-epidemic 4.2 (3.3, 5.3) | influenza, norovirus |
| Liu 2022 | Multiple | Four types of social contacts were identified: no contact, nonphysical contacts (e.g., in the physical presence of another person without face-to-face conversation or skin-to-skin touching or other indirect physical contacts), physical contacts (e.g., having a face-to-face conversation or skin-to-skin touching or other indirect physical contacts), and both nonphysical and physical contacts. | U.K. only shopping sites: Covid mean 1.83, flu 2.24; amusement parks: Covid 1.91, flu 2.47; animal attractions: Covid 1.72, flu 2.47; cultural activity sites: Covid 2.09, flu 2.76; outdoor sports sites: Covid 2.00, flu 2.43; exhibition venues: Covid 1.86, flu 2.56; sports centers: Covid 1.82, flu 2.49; entertainment venues: Covid 1.98, flu 2.84; religious sites: Covid 2.89, flu 2.24; | influenza |
| McCaw 2010 | Australia | Those involving a two-way or small group conversational exchange of at least 3 words, or any skin-to-skin contact | mean paper Friday 25.7, Sunday 21.2, Wednesday 27.5; PDA Friday 24.4, Sunday 20.3, Wednesday 21.8 | influenza |
| McCreesh 2022 | South Africa | Face-to-face conversation | 7.5 per day | influenza |
| Melegaro 2017 | Zimbabwe | An interaction between two individuals, either physical (when involving skin-to-skin contact), or non-physical (when involving a two-way conversation with three or more words in the physical presence of another person, but no skin-to-skin contact) | Mean 11.1 (median 9, IQR 6–14) | influenza |
| Mossong 2008 | Multiple | Either skin-to-skin contact such as a kiss or handshake (a physical contact), or a two-way conversation with three or more words in the physical presence of another person but no skin-to-skin contact (a nonphysical contact | Mean 13.4 | influenza |
| Munasinghe 2019 | Japan | An exchange of three Japanese sentences or a physical touch on the skin |  | influenza, norovirus |
| Nagpal 2024 | India | A face-to-face conversation within a distance of three feet, which may or may not have involved physical touch | winter: median 17 (IQR 12-26); summer: 15 (11-22); monsoon 14 (10-20) | influenza, norovirus |
| Neal 2020 | Fiji | Physical contact: Skin-to-skin contact, Non-physical contact: All other contact in the physical presence of another person, without skin-to-skin contact | Mean 6.40 (95% CI 6.24, 6.55) | norovirus |
| Nelson 2022 | United States | A physical contact as any contact involving physical touch, such as a handshake, hug, or kiss and a non-physical contact by an interaction in which the participant was within 6 feet of the other person and exchanged three or more words | Baseline: mean 13.9 (IQR 2, 10);  Follow-up: 14.5 (2, 11) | influenza, norovirus |
| Oguz 2018 | Turkey | An interaction in close proximity with three or more words directed to the infant or a physical skin-to-skin contact between infant and another person | Mean 4.6 ± 2.2 (range 1–18) | influenza, norovirus |
| Oh 2020 | South Korea | Physical contact was defined as skin-to-skin contact, such as a handshake or a hug, and non-physical contact constituted conversations of more than 2 words or 3 words within 1-2 m of another individual | Mean 6.6 (range 0-18) | influenza, norovirus |
| Oh 2020 | South Korea | Physical contact was defined as direct skin-to-skin contact (e.g., shaking hands, hugging, or kissing with the caregiver or friends), while non-physical contact was defined as engaging in a conversation of at least 2-3 words and keeping a distance of 1-2 meters from the other person (excluding nonverbal exchanges or short one-word conversations) | Mean 12.1 (SD 9.1) | influenza, norovirus |
| Oh 2021 | Korea | Physical contact referred to skin contact, such as shaking hands, hugging, and kissing; non-physical contact indicated conversations within 1 to 2 meters with more than 2 to 3 words spoken to each other | Mean 11.3 (SD ± 6.9, range 3–28) | influenza |
| Quaife 2020 | Kenya | Someone respondents met in person and with whom they had either (i) “physical contact (any sort of skin-to-skin contact e.g. a handshake, embracing, kissing, sleeping on the same bed/mat/blanket, sharing a meal together out of the same bowl, playing football or other contact sports, sitting next to someone while touching shoulder to shoulder, etc.”, or (ii) “Non-physical contact (you did not touch the person, but exchanged at least a few words, face-to-face within 2 metres - for example someone you bought something from in the market, or rode with on a minibus, or worked with in the same area) | Mean 18, median 13 (IQR 7-23) | influenza, norovirus |
| Read 2014 | China | Face-to-face conversation or skin-on-skin touch |  | influenza, norovirus |
| Smith 2022 | UK | Shopping for groceries/pharmacy, shopping for other items, providing help or care for a vulnerable person, meeting up with friends or family that they did not live with, going to a restaurant, café or pub, using public transport or a taxi/minicab and going out to work (number of days) |  |  |
| Stein 2014 | Multiple | A person sitting or standing within arm’s length of the participant (denoted as ‘YourSpace’) for 30 seconds or longer | Mean degree (variance)  Netherlands 25.6 (2212.2); Thailand 58.5 (8601.9) | influenza |
| Strömgren 2017 | Sweden | Physical touch or meeting places |  | norovirus |
| Sypsa 2021 | Greece | Either skin-to-skin contact or a 2-way conversation with 3 words spoken in the physical presence of another person | Before lockdown mean (95% CI): 20.7(18.9-22.5) During lockdown: 2.9(2.6-3.2) | influenza, norovirus |
| Taube 2024 | United States | A conversation lasting more than 5 minutes with a person who is closer than 6 feet away from you, or physical contact like hand-shaking, hugging, or kissing |  | influenza, norovirus |
| Thindwa 2022 | Malawi | Physical (participant’s skin to skin touch with a contact) and non-physical contacts (participant’s two-way close verbal conversation lasting for ≥5 min and with ≥3 words exchanged with a contact) | Mean 10.43 (quartiles 7,10,13) | influenza, norovirus |
| Tomori 2021 | Germany | People who you met in person and with whom you exchanged at least a few words, or with whom you had physical contact | wave 1 mean 2.0 (SD 1.9); wave 2 3.3 (4.7); wave 3 6.2 (18.4); wave 4 6.9 (326.3) | influenza, tuberculosis, norovirus |
| Trentini 2022 | Italy | A physical interaction or a two-way conversation of at least five words in the physical presence of another person | Mean 4.54 (95% CI 4.32-4.76) | influenza, norovirus |
| Tsuzuki 2022 | Japan | 1) A conversation of three or more sentences within two meters distance, 2) a direct conversation with others (indirect ones such as via telephone were excluded), 3) conversations with face coverings or partitioning, 4) a dinner with other people, where all those present at the table are considered contacts, 5) more than one conversation with the same person (counted as one contact), and 6) physical contact with a person (counted as one contact) | Median 3 (IQR 1-6), mean 8.92 (SD 25.45) | influenza |
| Tydeman 2023 | UK | Visits to shops, visits to other indoor public places, indoor visits to/from other households and public transport use |  |  |
| vanHoek 2013 | UK | An interaction in close proximity with three or more words directed to the infant or a physical skin-to-skin contact between infant and another person | Mean 6.68 (range 1-19) | influenza, norovirus |
| vanZandvoort 2022 | Somaliland | Any individual who was met in person during the recall period, and with whom the participant had at least a short conversation in short proximity | Mean (95% CI)  <2: 11.4 (10.4-12.4); 2-5: 11.7 (10.8–12.6); 6-14: 3.0 (11.7–14.4); 15-29: 14.3 (12.9-15.8); 30-49: 14.6 (12.6-16.6); 50+: 12.3 (11-13.6) | influenza, norovirus |
| Vino 2017 | Australia | Who slept in your house last night? | Median 6 (range 1-23) |  |
| Wang 2024 | China | Either physical contact such as shaking hands or hugging, or a two-way conversation with three or more words in the physical presence of another person | Mean 11.51 (SD 5.96) | influenza, norovirus |
| Watson 2017 | Fiji | Shared meals (lunch and dinner) | iTaukei median 4 (IQR 2-7); non-iTaukei 3 (2-5) |  |
| Wong 2023 | Multiple | Anyone who met the participant in person with whom at least a few words were exchanged or physical contact was made | UK mean (95% CI): 3.22 (3.19–3.26); Belgium: 3.94 (95%CI=3.85–4.03); Netherlands: 3.63 (95%CI=3.52–3.74); Germany: 2.64 (95%CI=2.58–2.70); Malta: 7.25(6.78–7.71); Austria: 2.81(2.65–2.97) | influenza, norovirus |
| Zhang 2019 | China | Either, (1) a two-way conversation with three or more words in the physical presence of another person (conversational contact), or (2) physical skin-to-skin contact (e.g. a handshake, hug, kiss or contact sports) | Median 10 | influenza, norovirus |
| Zhang 2020 | China | Either a two-way conversation involving three or more words in the physical presence of another person or a direct physical contact (e.g., a handshake) | outbreak period: mean 1.9 post-lockdown period: mean 3.6 | influenza, norovirus |
| Zhao 2022 | China | (1) A two-way conversation that involved at least 3 words in the physical presence of another person (conventional contact), or (2) a direct physical contact (e.g., a handshake, hug, kiss) | Mean 2.3, median 2 (IQR 1.0-3.0) | influenza, norovirus |
| Eames 2010 | UK | Either talking face-to-face or skin-to-skin contact (e.g. a handshake, a kiss, contact sports) | Initial survey mean 3.58 (SD 3.75); follow-up 10.30 (8.51) | influenza |
| Potter 2019 | Senegal | Speaking with a person in the same location | Mean degree (95% CI)  asymptomatic morning 16.5 (14.3, 18.7); symptomatic morning 15.0 (12.9, 17.1); asymptomatic afternoon 14.8 (12.7, 16.9); symptomatic afternoon 13.5 (11.5, 15.4) | influenza, NA, NA |
| Bridgen 2023 | UK | Someone whom the participant had a face-to-face conversation with | Mean customer contacts 71.6 (95% CI 61.0 - 84.1); depot contacts 15.0 (11.2 to 19.2) | influenza |
| Chen 2012 | Taiwan | A 2-way conversation (at a distance that did not require raised voices) in which at least 3 words were spoken by each party, and in which there was no physical barrier between the 2 parties (such as a security screen). In addition, the conversation distance had to be less than 1 meter. | Mean (SD)  Grade 7: 11.18 (7.98) Grade 8: 10.03 (7.54) Grade 9: 9.44 (8.68) | influenza, norovirus |
| Chen 2015 | Taiwan | A two-way conversation in which at least three words were spoken by each party. Two types of physical contact were defined: (i) two-way conversations during which at least three words were spoken (conversation only), and (ii) contacts which involved any sort of skin-to-skin contact (physical contact) | Term time: mean 20.0 (SD 11.7) Holiday period: 12.6 (10.7) | influenza, norovirus |
| Cohen 2012 | United States | Each time someone entered an observed patient’s room (or, in the case of shared rooms, a patient’s area, as indicated by curtain dividers), the observer recorded the person’s role in patient care, times of room entry and exit, and highest level of contact made in the room (touched nothing, touched patient’s environment only, touched patient’s intact skin only, touched patient’s blood or body fluids, or unknown) | Medical staff: mean 2.8 (range 0.5-7.0); nursing staff 4.5 (0.5-18.0) contacts per hour |  |
| Conlan 2011 | UK | Which pupils in your class do you spend the most time with? Which pupils in other classes in the school do you spend the most time with? |  |  |
| Eames 2011 | UK | Talking face-to-face or skin-to-skin contact (e.g. a handshake, a kiss, contact sports, etc.) | term mean 19.0 (SD 8.9); holiday 9.4 (6.0) | influenza, norovirus |
| Fairbanks 2023 | UK | Participation in the setting, the number of distinct activities, total contacts (the sum of the mid-points of estimated contacts during each activity), total duration of activities, and person-contact-hours (PCH) calculated as the summed product of the midpoint of the estimated contacts and the duration (in hours) of each activity, the total contacts not including the participant’s household members and PCH not including the participant’s household members | Mean (SD)  household 3.02 (2.23); abroad 7.02 (28.26); campus 2.40 (9.77); exercise 4.88 (15.29); hospitality 4.63 (21.37); non-university work 4.44 (20.07); non-private travel 9.04 (37.54); other 3.99 (12.85); research 6.38 (16.95); retail 41.33 (56.47); social 1.61 (4.21); teaching 9.08 (27.10); testing 1.52 (3.06) |  |
| Glass 2008 | United States | Within 3 ft and for a recognizable length of time | Mean contact-level-hours per person per day (coefficient of variation)  5th grade: 163.46 (0.32); 7th grade: 130.96 (0.39); 9-10th grade: 149.16 (0.69); 11-12th grade: 145.52 (0.51) | influenza, norovirus |
| Grantz 2021 | United States | Any individual they talked with, played with, or touched | Mean 14.2 (SD 9.4) | influenza |
| Gravagna 2022 | United States | ≥ 15 minutes of close contact with people not living in the household | Median (IQR)  Wave1: 1 (0–3), Wave2: 2 (1–6), Wave3: 5 (2–10), Wave4: 5 (2–10), |  |
| Jackson 2011 | UK | Spoken to | typical school day: mean 70.3 (SD 40.8); school closure: 24.8 (SD 22.5) | influenza |
| Johnson 2024 | United States | Having a face-to-face conversation, skin-to-skin contact, indoor in a room for 10+ minutes, or non-socially distanced indoors or outdoors for 10+ minutes. | Median 3 for Virtual Learning (VL) period 7 for In-Person Masking (IPM) period | influenza, norovirus |
| Kiti 2023 | United States | Proximate (no conversation and no physical contact but within 6 feet of another person for more than 20 s, e.g., sitting next to someone in public transport or standing in line), conversational (a two-way conversation with three or more words exchanged in the physical presence of another person), or physical (directly touching someone (skin-to-skin contact) or the clothes they are wearing, intentionally or unintentionally, including a handshake, fist bump, elbow bump, foot bump, hug, and kiss) | Median (IQR) contacts over both days: R1: 2 (1–4); R2: 7 (4–10); R3: 7 (4–12); R4: 8 (4–13) Mean (SD) contacts over both days: R1: 2.5 (0.2); R2: 8.2 (0.9); R3: 9.2 (0.5); R4: 10.1 (0.7) | influenza, norovirus |
| Kucharski 2018 | UK | Conversation |  |  |
| Leecaster 2016 | United States | People they talked to or touched |  | influenza |
| Litvinova 2019 | Russia | A two-way conversation of at least 5 words in the physical presence of another person | school/class open: mean 12.6(95% CI 11.7-13.4) school/class closed: 7.4(6.7-8.1) | influenza |
| Luh 2016 | Taiwan | (i) Two-way conversations during which at least three words were spoken (conversation only), and (ii) contacts that involved any sort of skin-to-skin contact (physical contact) | Flu season: median 12 (IQR 7-21.5), mean 16.3 (SD 12.9); non-flu season 12.5 (6.5-21), 14.6 (9.5) | influenza |
| Potter 2015 | Switzerland | Either conversation held at <2 m distance and with more than ten words spoken, or any sort of physical contact with another individual |  | influenza |
| Powers 2022 | United States | People they encountered at <6 feet for each location where they reported spending ≥ 15 minutes |  | influenza, tuberculosis |
| Smieszek 2012 | Switzerland | (i) A conversation held at <2 m distance and with more than ten words spoken, or as (ii) any sort of physical contact with another participant in the study |  | influenza |
| Smieszek 2014 | United States | A person with whom the participant had one or more interactions that (i) were a maximum of two arms-lengths apart, (ii) more than a 10-word conversation, and (iii) occurred only while at school | Mean degree  March 14 : 8.0; March 15: 10.4; March 16: 5.3; | influenza |
| Trickey 2021 | UK | Individual contacts: spoke to in person one-on-one, including those in their household and support bubble. Other contacts: spoke in person to many people one-on-one in the same setting (but they did not have the opportunity to speak to each other) | Staff rule-of-six median 3 (IQR 1-5); Staff lockdown 2 (1-3); students rule-of-six 3 (1-6); students lockdown 2 (1-4) | influenza |
| Yang 2022 | United States | The people the participants meet at a distance of less than 2 m |  | influenza |
| You 2013 | Taiwan | A 2-way conversation (at a distance that did not require raising the voice) in which at least 3 words were spoken by each party and in which there was no physical barrier between the 2 parties (such as security screens). The conversation distance was less than 1 m. | Grade 7: mean 9.44 ± SD 8.68 Grade 8: 10.12 ± 4.5 Grade 9: 11.18 ± 7.98 | influenza |
| Zheng 2022 | China | A two-way conversation with three or more words in the physical presence of another person (conversational touch), or skin-to-skin contact (such as a handshake, hug, kiss, or contact sport) | individual mean 3.14 (95% CI 3.13–3.15); group 37.90 (37.20-38.70) | influenza, tuberculosis, norovirus |
| Zissette 2023 | United States | Any two-way conversation with an exchange of three or more words in the physical presence (i.e., someone close enough to touch) of another person | mean 9, median 10 | influenza, norovirus |
