# Supplementary material for: Relevance of social contact definitions for use in infectious disease transmission modeling: a systematic review and recommendations
Source: BMC Infect Dis. 2026 Mar 18;26:836. doi: 10.1186/s12879-026-12938-y (PMC13112660; doi:10.1186/s12879-026-12938-y)
Supplement: Supplementary file 5 — Supplementary Material 5: Appendix D [file 12879_2026_12938_MOESM5_ESM.docx]

**Appendix D**

**Mixed Methods Appraisal Tool Assessment**

**Table S2**. Summary of consensus results of the Mixed Methods Appraisal Tool (MMAT) risk of bias assessment.

| **Criterion** | **Yes***^1^* | **No***^1^* | **Can’t tell***^1^* |
| --- | --- | --- | --- |
| Clear research question(s) | 112 (100%) | 0 (0%) | 0 (0%) |
| Data appropriate to answer research question(s) | 111 (99%) | 1 (0.9%) | 0 (0%) |
| Sampling strategy appropriate | 106 (95%) | 1 (0.9%) | 5 (4.5%) |
| Study population is representative of source population | 46 (41%) | 21 (19%) | 45 (40%) |
| Contact measures are appropriate | 110 (98%) | 1 (0.9%) | 1 (0.9%) |
| Risk of non-response bias is low | 23 (21%) | 19 (17%) | 70 (63%) |
| Analysis methods are appropriate | 109 (97%) | 2 (1.8%) | 1 (0.9%) |
| *^1^*n (%) | |  |  |

**Table S3**. Individual-level consensus results of the Mixed Methods Appraisal Tool (MMAT) risk of bias assessment.

| **Study ID** | **Clear research questions** | **Collected data allow to address the research questions** | **Sampling strategy is relevant to address the research question** | **Sample is representative of the target population** | **Measurements are appropriate** | **Risk of nonresponse bias is low** | **Statistical analysis is appropriate to answer the research question** |
| --- | --- | --- | --- | --- | --- | --- | --- |
| Backer 2024 | Yes | Yes | Yes | Yes | Yes | Can't tell | Yes |
| Bosetti 2021 | Yes | Yes | Yes | Can't tell | Yes | Can't tell | Yes |
| Drolet 2022 | Yes | Yes | Yes | Yes | Yes | Can't tell | Yes |
| Johnson 2024 | Yes | Yes | Yes | Can't tell | Yes | No | Yes |
| Kurahashi 2023 | Yes | Yes | Can't tell | Can't tell | Yes | Can't tell | Yes |
| Leach 2023 | Yes | Yes | Yes | No | Yes | Can't tell | Yes |
| McCreesh 2016 | Yes | Yes | Yes | Yes | Yes | Can't tell | Yes |
| Pilny 2021 | Yes | Yes | Yes | Can't tell | Yes | Can't tell | Yes |
| Stein 2014 | Yes | Yes | Yes | No | Yes | Can't tell | Yes |
| Taube 2024 | Yes | Yes | Yes | Yes | Yes | Can't tell | Yes |
| Trickey 2021 | Yes | Yes | Yes | Can't tell | Yes | Can't tell | Yes |
| Vino 2017 | Yes | Yes | Yes | Can't tell | Yes | Can't tell | Yes |
| Yang 2024 | Yes | Yes | Yes | Yes | Yes | No | Yes |
| Zhang 2019 | Yes | Yes | Yes | Yes | Yes | Can't tell | Yes |
| Zhang 2020 | Yes | Yes | Yes | No | Yes | Can't tell | Yes |
| Ajelli 2017 | Yes | Yes | Can't tell | No | Yes | Can't tell | Yes |
| Gravagna 2022 | Yes | Yes | Yes | No | Yes | Can't tell | Yes |
| Munasinghe 2019 | Yes | Yes | Yes | Yes | Yes | Can't tell | Yes |
| Talcott 2022 | Yes | No | Yes | Can't tell | Yes | No | No |
| Luh 2016 | Yes | Yes | Yes | Can't tell | Yes | No | Yes |
| Wang 2024 | Yes | Yes | Yes | Can't tell | Yes | Can't tell | Yes |
| Glass 2008 | Yes | Yes | Yes | Can't tell | Yes | Can't tell | Yes |
| Zheng 2022 | Yes | Yes | Yes | Yes | Yes | Can't tell | Yes |
| Eames 2011 | Yes | Yes | Can't tell | Can't tell | Yes | No | Yes |
| Oh 2020 | Yes | Yes | Yes | No | Yes | Can't tell | Yes |
| Liang 2023 | Yes | Yes | Yes | Yes | Yes | Can't tell | Yes |
| Smieszek 2014 | Yes | Yes | Yes | Yes | Yes | Yes | Yes |
| Dodd 2016 | Yes | Yes | Yes | Yes | Yes | Yes | Yes |
| Ibuka 2016 | Yes | Yes | Yes | Yes | Yes | Yes | Yes |
| McCaw 2010 | Yes | Yes | Yes | No | Yes | Can't tell | Yes |
| lePolaindeWaroux 2018 | Yes | Yes | Yes | Yes | Yes | Yes | Yes |
| English 2018 | Yes | Yes | Yes | Can't tell | Yes | Can't tell | Yes |
| Liu 2022 | Yes | Yes | Yes | Yes | Can't tell | Can't tell | Yes |
| Neal 2020 | Yes | Yes | Yes | No | Yes | Can't tell | Yes |
| Andrejko 2022 | Yes | Yes | Yes | Yes | Yes | Can't tell | Yes |
| Jackson 2011 | Yes | Yes | Can't tell | No | Yes | Yes | Yes |
| You 2013 | Yes | Yes | Yes | Can't tell | Yes | Can't tell | Yes |
| Eames 2010 | Yes | Yes | Yes | Yes | Yes | No | Yes |
| Wong 2023 | Yes | Yes | Yes | Yes | Yes | Can't tell | Yes |
| Tomori 2021 | Yes | Yes | Yes | No | Yes | Can't tell | Yes |
| Hoang 2021 | Yes | Yes | Yes | Yes | Yes | Can't tell | Yes |
| Smith 2020 | Yes | Yes | Yes | Can't tell | Yes | No | Yes |
| Zissette 2023 | Yes | Yes | Yes | Can't tell | Yes | Yes | Yes |
| Potter 2015 | Yes | Yes | Yes | No | Yes | Yes | Yes |
| Quaife 2020 | Yes | Yes | Yes | Yes | Yes | Can't tell | Yes |
| Bridgen 2023 | Yes | Yes | Yes | Yes | Yes | No | Yes |
| Dobreva 2022 | Yes | Yes | Yes | Yes | Yes | Yes | Yes |
| Chen 2012 | Yes | Yes | Yes | Can't tell | Yes | Yes | Yes |
| Oh 2020 | Yes | Yes | Yes | No | Yes | Can't tell | Yes |
| Fu 2020 | Yes | Yes | Yes | Can't tell | Yes | Can't tell | Yes |
| Sypsa 2021 | Yes | Yes | Yes | Can't tell | Yes | Can't tell | Yes |
| Zhao 2022 | Yes | Yes | Can't tell | Can't tell | Yes | Yes | Yes |
| Auranen 2021 | Yes | Yes | Yes | Can't tell | Yes | Can't tell | Yes |
| Cohen 2012 | Yes | Yes | Yes | Can't tell | Yes | Can't tell | Yes |
| Powers 2022 | Yes | Yes | Yes | Yes | Yes | No | Yes |
| Smieszek 2012 | Yes | Yes | Yes | Yes | Yes | Can't tell | Yes |
| Tydeman 2023 | Yes | Yes | Yes | Can't tell | Yes | Yes | Yes |
| Feehan 2021 | Yes | Yes | Yes | Yes | No | Can't tell | Can't tell |
| Tizzani 2023 | Yes | Yes | Yes | Yes | Yes | Can't tell | Yes |
| Oh 2021 | Yes | Yes | Yes | No | Yes | Can't tell | Yes |
| Kleynhans 2021 | Yes | Yes | Yes | Can't tell | Yes | Yes | Yes |
| Chen 2015 | Yes | Yes | Yes | Can't tell | Yes | No | Yes |
| Oguz 2018 | Yes | Yes | Yes | Yes | Yes | Yes | Yes |
| DeStefano 2011 | Yes | Yes | Yes | No | Yes | No | Yes |
| Johnstone-Robertson 2011 | Yes | Yes | Yes | Yes | Yes | Can't tell | Yes |
| Conlan 2011 | Yes | Yes | Yes | Can't tell | Yes | Can't tell | No |
| Backer 2023 | Yes | Yes | Yes | Yes | Yes | No | Yes |
| McCreesh 2022 | Yes | Yes | Yes | Yes | Yes | Yes | Yes |
| Brankston 2021 | Yes | Yes | Yes | Yes | Yes | Can't tell | Yes |
| Grantz 2021 | Yes | Yes | Yes | Yes | Yes | Yes | Yes |
| Strömgren 2017 | Yes | Yes | Yes | No | Yes | No | Yes |
| Tsuzuki 2022 | Yes | Yes | Yes | Can't tell | Yes | Can't tell | Yes |
| Leung 2023 | Yes | Yes | Yes | Yes | Yes | Can't tell | Yes |
| Huang 2020 | Yes | Yes | Yes | Can't tell | Yes | Can't tell | Yes |
| Leung 2017 | Yes | Yes | Yes | No | Yes | Can't tell | Yes |
| Litvinova 2019 | Yes | Yes | Yes | Yes | Yes | Can't tell | Yes |
| Stefkovics 2024 | Yes | Yes | Yes | Yes | Yes | Can't tell | Yes |
| DelFava 2021 | Yes | Yes | Yes | No | Yes | Can't tell | Yes |
| Hidano 2022 | Yes | Yes | Yes | Can't tell | Yes | Can't tell | Yes |
| Beale 2022 | Yes | Yes | Yes | Can't tell | Yes | No | Yes |
| Thindwa 2022 | Yes | Yes | Yes | Yes | Yes | Yes | Yes |
| Trentini 2022 | Yes | Yes | No | Can't tell | Yes | Can't tell | Yes |
| Nelson 2022 | Yes | Yes | Yes | Yes | Yes | No | Yes |
| vanZandvoort 2022 | Yes | Yes | Yes | Can't tell | Yes | Can't tell | Yes |
| Fairbanks 2023 | Yes | Yes | Yes | Can't tell | Yes | Can't tell | Yes |
| Kiti 2023 | Yes | Yes | Yes | Yes | Yes | Can't tell | Yes |
| Danon 2013 | Yes | Yes | Yes | No | Yes | No | Yes |
| Read 2014 | Yes | Yes | Yes | Yes | Yes | Can't tell | Yes |
| Kwok 2014 | Yes | Yes | Yes | Yes | Yes | Can't tell | Yes |
| Goeyvaerts 2018 | Yes | Yes | Yes | Can't tell | Yes | Can't tell | Yes |
| Yang 2022 | Yes | Yes | Yes | No | Yes | No | Yes |
| Mossong 2008 | Yes | Yes | Yes | Yes | Yes | Can't tell | Yes |
| Higgins 2023 | Yes | Yes | Yes | Can't tell | Yes | Can't tell | Yes |
| Horby 2011 | Yes | Yes | Yes | Can't tell | Yes | Can't tell | Yes |
| Wood 2012 | Yes | Yes | Yes | Can't tell | Yes | Can't tell | Yes |
| Fu 2012 | Yes | Yes | Yes | Yes | Yes | Can't tell | Yes |
| Bridgen 2022 | Yes | Yes | Yes | No | Yes | Can't tell | Yes |
| Smith 2022 | Yes | Yes | Yes | Yes | Yes | Can't tell | Yes |
| vanHoek 2013 | Yes | Yes | Yes | Can't tell | Yes | Yes | Yes |
| Ferraro 2014 | Yes | Yes | Yes | Can't tell | Yes | Yes | Yes |
| Kiti 2014 | Yes | Yes | Yes | Can't tell | Yes | Yes | Yes |
| Grijalva 2015 | Yes | Yes | Yes | Can't tell | Yes | Yes | Yes |
| Beraud 2015 | Yes | Yes | Yes | Yes | Yes | Can't tell | Yes |
| Leecaster 2016 | Yes | Yes | Yes | Yes | Yes | Yes | Yes |
| Melegaro 2017 | Yes | Yes | Yes | Yes | Yes | Can't tell | Yes |
| Watson 2017 | Yes | Yes | Yes | Can't tell | Yes | Yes | Yes |
| Kucharski 2018 | Yes | Yes | Yes | Can't tell | Yes | No | Yes |
| Kumar 2018 | Yes | Yes | Yes | Can't tell | Yes | Yes | Yes |
| Ghilotti 2019 | Yes | Yes | Yes | Yes | Yes | No | Yes |
| Potter 2019 | Yes | Yes | Yes | No | Yes | Can't tell | Yes |
| Latsuzbaia 2020 | Yes | Yes | Yes | Can't tell | Yes | Can't tell | Yes |
| Nagpal 2024 | Yes | Yes | Yes | Can't tell | Yes | Can't tell | Yes |
